# Musculoskeletal research in human space flight – unmet needs for the success of crewed deep space exploration

**DOI:** 10.1038/s41526-023-00258-3

**Published:** 2023-01-28

**Authors:** Anna-Maria Liphardt, Rodrigo Fernandez-Gonzalo, Kirsten Albracht, Jörn Rittweger, Laurence Vico

**Affiliations:** 1 Department of Internal Medicine – Rheumatology & Immunology, Universitätsklinikum Erlangen & Friedrich-Alexander-Universität Erlangen-Nürnberg, Ulmenweg 18, 91054 Erlangen, Germany; 2 Deutsches Zentrum Immuntherapie (DZI), Universitätsklinikum Erlangen & Friedrich-Alexander-Universität Erlangen-Nürnberg, Ulmenweg 18, 91054 Erlangen, Germany; 3 Department of Laboratory Medicine, Division of Clinical Physiology, Karolinska Institutet, Alfred Nobels Allé 8, 14152 Huddinge, Sweden; 4 Unit of Clinical Physiology, Karolinska University Hospital, Hälsovägen 13, 14157 Huddinge, Sweden; 5 Faculty of Medical Engineering and Technomathematics, University of Applied Sciences Aachen, Campus Jülich, Heinrich-Mußmann-Str. 1, Jülich, Germany; 6 Institute of Movement and Neurosciences, German Sport University Cologne, Am Sportpark Müngersdorf, Cologne, Germany; 7 Institute of Aerospace Medicine, German Aerospace Center (DLR), Linder Höhe, Cologne, Germany; 8 Department of Pediatrics and Adolescent Medicine, University Hospital Cologne, Cologne, Germany; 9 Univ. Jean Monnet, Mines Saint-Étienne, INSERM, U 1059 Sainbiose, 42023 Saint-Étienne, France

**Keywords:** Translational research, Predictive markers, Diagnostic markers

## Abstract

Based on the European Space Agency (ESA) Science in Space Environment (SciSpacE) community White Paper “Human Physiology – Musculoskeletal system”, this perspective highlights unmet needs and suggests new avenues for future studies in musculoskeletal research to enable crewed exploration missions. The musculoskeletal system is essential for sustaining physical function and energy metabolism, and the maintenance of health during exploration missions, and consequently mission success, will be tightly linked to musculoskeletal function. Data collection from current space missions from pre-, during-, and post-flight periods would provide important information to understand and ultimately offset musculoskeletal alterations during long-term spaceflight. In addition, understanding the kinetics of the different components of the musculoskeletal system in parallel with a detailed description of the molecular mechanisms driving these alterations appears to be the best approach to address potential musculoskeletal problems that future exploratory-mission crew will face. These research efforts should be accompanied by technical advances in molecular and phenotypic monitoring tools to provide in-flight real-time feedback.

## Introduction

The European Space Agency (ESA) Science in Space Environment (SciSpacE) community White Paper “Human Physiology – Muskuloskeletal system” serves as a basis for this perspective (https://esamultimedia.esa.int/docs/HRE/12_HumanResearch_HumanPhysiology.pdf). The reader must be aware that the purpose of this article is not to review all published results, but rather to highlight unmet needs and suggest new avenues for future studies.

The musculoskeletal system is essential for sustaining physical function and energy metabolism, and it is becoming obvious that the maintenance of health during exploration missions, and consequently mission success, will be tightly linked to the ability to perform efficient movements with all body segments. The known effects of reduced gravitational forces and mechanical loads on bone and muscle comprise overall loss of mass in both tissues, resulting in site-dependent altered biomechanical and endocrine functions, deterioration of bone integrity and bone strength. These changes have been summarized elsewhere^1–4^. Potential alterations in tissue composition and mechanical properties of articular cartilage, tendon, ligament, and connective tissue during spaceflight are currently not well understood^5,6^ because they are not relevant to mission success at current mission lengths of up to six months. In light of upcoming multiyear missions, the response of these relatively slow-adapting tissues and their role in maintaining crew health and musculoskeletal function needs to be addressed.

The high degree of cross-talk between the musculoskeletal system and other organs^7^ is an important aspect to consider for successful crewed deep space exploration. The phenotypic changes in the musculoskeletal system originate from molecular events that are altered by space stressors (i.e., microgravity, radiation, psychological stress and confinement). Although research has begun to uncover the link between these molecular events and the corresponding phenotypic outcomes^8^, a better understanding is needed to determine the multi-organ alterations and thus develop effective countermeasures.

Some of the countermeasures used to date to offset space-induced musculoskeletal alterations have shown promising results. However, for missions leaving low Earth orbit (LEO), improvements in individualization, efficacy, hardware (i.e., dimensions), and adaptability are required^9^. It follows that the advantages and disadvantages of countermeasures should be explored with the goal of integrating interventions that positively impact multiple systems simultaneously.

A major gap is the need to integrate data from different areas of human space life sciences, as well as space agencies’ management plans with respect to research priorities for exploration missions. While the degree of integration of different disciplines and standardization of experimental conditions and protocols in space research is very high, especially for ground-based analog studies, there is a lag in planned and shared data analysis. Thus, integrative data analysis and interpretation of the results need to be enabled, with the goal of understanding how to maintain physiological function, and thus crew health, during and after space missions at a level that ensures mission success, which may not necessarily be similar to what is required on Earth (i.e., 1 G).

In this context, the observed interindividual variability^10–13^ should be a focus in future study designs to allow personalization of countermeasure regimens^14^. Indeed, factors such as age and sex (e.g., hormone levels) need to be investigated more closely to identify elements that may drive and/or predict variation in musculoskeletal adaptations to immobilization or space environments^11^. A major milestone for studying interindividual differences in musculoskeletal response to spaceflight would be for space agencies to guarantee raw data from analog and space studies in open data repositories (ideally as a joined effort of all agencies).

Studies of space analogs in human, animal, in silico, or in vitro models are important complements to flight studies because they can provide fundamental understanding of underlying mechanisms toward the overall goal of mitigating the effects of prolonged spaceflight on the musculoskeletal system before we expand our exploration efforts beyond LEO^15–17^. In this context, research proposals that simultaneously cover basic science, analog, and spaceflight settings using translational research could be promoted^18^.

**Fig. 1:**
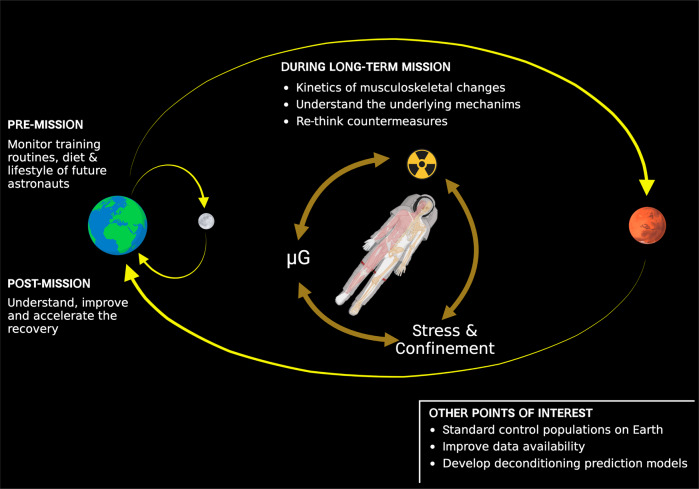
Crewed deep space exploration. Unmet needs and points of interest to maintain musculoskeletal health and performance during upcoming human long-term space missions.

Space-related musculoskeletal research should not focus solely on the “in-flight” period. The benefits of understanding musculoskeletal dynamics before and after flight must be highly considered, as this can provide important clues for understanding musculoskeletal adaptation during long-term spaceflight. (Fig. 1). Especially, data from the recovery period will be valuable for mathematical models of tissue adaptation. To further complicate the matter, exploration missions will include flight periods interrupted by a phase of gravitational load (on Moon or Mars) during which the crewmembers must physically work. This will require different functionality of the musculoskeletal system than that which we know from landings on Earth, where the crew is surrounded by support personnel with a recovery program in place.

With this in mind, as part of the update of the ESA’s SciSpacE scientific community white papers, the authors of this article have developed a list of suggestions for ensuring musculoskeletal health and performance during long-term space missions. These suggestions are summarized below.

## Targeted periods of observation

### Pre-flight

Monitoring training routines, diet, lifestyle habits, and health status before the mission (pre-flight) is a challenging and demanding task that can present numerous logistical difficulties. However, important information for understanding and predicting individual differences in musculoskeletal adaptation during space missions may reside in the general daily routines of the crew as well as in baseline characteristics (e.g., biomarkers of tissue turnover, phenotypic characteristics of muscle and bone, etc.)^19,20^. Therefore, efforts should be made to wisely collect potentially important information during this period.

### In-flight

For in-flight data collection, there are three different aspects that need to be considered in the context of crewed exploration missions.

#### Kinetics of musculoskeletal changes in space

The longer exploration space missions will bring new challenges to musculoskeletal health. One of the first steps to be taken in the coming years is to understand whether the musculoskeletal adaptations observed on missions of several months^3,19–22^ can be extrapolated to space missions of more than twelve months. Recent studies that examined bone health during the recovery phase suggest that bone atrophy and functional decline persist well beyond return from space^20,22^. This information should be considered in modeling approaches and suggests that bone tissue changes may not plateau after 6 months of spaceflight. It follows that during these long-term missions, quantification of musculoskeletal load should be achieved by integrating experimental (lower limb kinematics, kinetics, imaging data pre- and post-mission, and during exercise) and computational simulations, as this would allow tissue deterioration to be matched to the expected occurring loads. Ultimately, in the context of astronauts spending years in space, it will be important to investigate whether it is necessary to set a limit on the length of time spent in space to prevent failure of essential musculoskeletal functions.

The multiple components of the musculoskeletal system likely suffer the space-induced alterations in a dissimilar chronological fashion. For example, events in tissues in which the adaptation appears to be less pronounced compared to skeletal muscle (e.g., cartilage, tendon, ligament) and which have not been well studied need to be investigated. Moreover, the regenerative capacity of these tissues is very limited, which also makes the treatment of potential injuries more challenging^23–25^. In this context, it is important to note that there is currently no human ground-based space analog that mimics missions of more than two months, and for certain organ systems, there is no comparable situation for healthy humans on Earth (e.g., immobilization for several years). Thus, close observations of the current crew are the only way to answer many of the open questions along with the systematic study of musculoskeletal adaptation at mission lengths longer than 6 months.

For crewed exploration missions, it will be essential to develop tools that allow real-time monitoring of individual musculoskeletal changes during flight. Better monitoring tools will help individualize countermeasures and anticipate dangerous levels of musculoskeletal alterations. This will require defining biomarkers that are indicative of musculoskeletal function. These could include coding and noncoding RNAs, proteins, metabolites, and imaging techniques, in combination with functional tests. In this context, high-throughput techniques may prove useful because they could define the molecular events driving the phenotypic alterations during spaceflight^26^. An important aspect to consider regarding the monitoring tools and the timing of musculoskeletal changes in space is to understand and determine the specific kinetics of the anabolic and catabolic biomarkers used. For example, countermeasures that are effective in preserving bone during bed rest engender their success not by suppressing bed rest-induced bone resorption, but rather by fostering bone formation to a level that negates the elevation in bone resorption, thereby equalizing bone balance^27,28^. If only bone resorption had been measured and not bone formation and content, then an effective countermeasure would have been discarded as ineffective. More generally, therefore, it seems imperative to jointly study tissue break-down, tissue de-novo formation and tissue abundance to obtain a complete understanding of the changes in bone, muscle, tendon, and all other constituents of the musculoskeletal system affected by spaceflight.

#### Understand the underlying mechanisms

The nature of current space studies is very often observational. This should evolve to more mechanistic research to understand the underlying mechanism and tissue specificity of the observed changes. While available methods for studying in-flight molecular changes are limited, efforts should be made to miniaturize and simplify innovative and rapid analysis techniques during space missions, along with pre- and post-mission experiments with a mechanistic approach.

Phenotypic adaptations of skeletal muscle to microgravity have been investigated for many years. These changes occur quite rapidly (i.e., days)^29^, are relatively easy to monitor, and adaptability is very high. However, the molecular events driving these alterations are less understood. Such information could be critical, for example, to provide personalized countermeasure programs and to improve countermeasure efficacy. Despite some inherent limitations, the multifaceted stressors of the space environment (low gravity, g transitions, psychological stress, confinement, radiation) could be investigated in isolation or combined using human analogs, and animal and in vitro models. The functional and molecular insights resulting from such studies could provide important clues to refine countermeasures for the stressors that more severely affect the musculoskeletal system during exploration missions, as well as to determine the weakest component of skeletal muscle. Furthermore, we do not know how long-term spaceflight affects the different cell subpopulations within the muscle and how the regenerative capacity of skeletal muscle may be altered^30^. Also, the role of neuromuscular alterations, including changes in neuromuscular junction, in the loss of muscle mass and function caused by long-term spaceflight should be investigated.

Bone adaptation has been studied for many years as part of spaceflight research. As with muscle, we know that bone loss is accelerated during spaceflight. Although osteoclastic bone resorption, as measured by biomarkers in serum or urine, increases with a half-life of 11 days or less^3^, bone turnover at the tissue level is slower compared with skeletal muscle, and it therefore takes longer for changes to become apparent at the structural level. With advancing imaging methods such as high-resolution peripheral quantitative computed tomography (HR-pQCT), new opportunities are emerging to understand the 3-dimensional microarchitecture of bone, including potential changes due to lack of gravity (i.e., lack of force vector guidance) and how this affects bone function (e.g., bone strength and fracture healing). In addition, it will be important to identify events associated with altered bone resorption and formation activities.

At the cellular level, despite initial attempts to define changes in bone marrow cell populations (e.g., immune and hematopoietic cells, mesenchymal stem cells, adipocytes)^31^, potential interactions between different cell types in response to the space environment remain to be investigated, as does the adaptation of osteocytes, the chief orchestrator of bone formation and resorption^32^.

One factor that has received little attention is the effect of fluid shift on bone deconditioning. Thus, it is currently unknown whether the redistribution of skeletal minerals is caused by fluid shift, load bearing, or both. In addition, while the flow-induced strain of the interstitial fluid in the lacuno-canalicular network in osteocytes has been modeled using numerical simulations^33^, there are still unanswered questions regarding the vascularisation of bone and how this relates to fluid shift.

Some tissues of the musculoskeletal system that have been barely investigated in the context of human spaceflight deserve special mention: Articular cartilage, tendons, ligaments, and connective tissue. These tissues have in common that their repair and regeneration capacities are limited compared to muscle and bone^34^.

Articular cartilage, and thus joint health, is essential for the ability to move. Cartilage degradation increases during immobilisation^35–39^, and cartilage atrophy and loss of function may become more significant with increasing mission duration. Clinical studies have shown cartilage atrophy as a result of immobilization^40^ or paralysis^41^, results supported by animal studies^42–50^. Its slow metabolism and lack of visible changes during the initial deterioration phases call for research defining appropriate biomarkers. Importantly, cartilage regeneration and repair capacity are limited, which requires a comprehensive definition of adequate countermeasures. Indeed, research on exercise countermeasures to prevent cartilage degeneration is inconclusive^51^, and current countermeasures investigated during bed rest seem unable to prevent the effects of immobilization on cartilage biomarkers^36–38^.

Astronauts frequently report low back pain during the initial phase of spaceflight and after flight. It has been hypothesized that the reduced spinal loading in space leads to intervertebral disc swelling (increase in hydration and pressure), which contributes to spinal lengthening during flight and increased risk of disc herniation^52^. It appears that changes in disc height and hydration are in fact negligible^53^. Recent findings suggest that atrophy of lumbar paraspinal muscles, together with vertebral bone and intervertebral disc changes, may play an important role in explaining the longitudinal mechanisms and risk factors for the development of back pain and disc herniation in astronauts^4^. This clearly illustrate the importance of considering the interrelationships between the various components of the musculoskeletal system.

Tendons, ligaments, and connective tissue provide the key connections between bone and muscle, playing an integral role in joint stability and the transmission of forces to the skeleton. Knowledge about the altered integrity of these tissues due to unloading and their recovery is based primarily on animal studies using ground-based analogs of microgravity^6,54–57^. Indeed, the effects of short- and long-term exposure to microgravity on tendons at the cellular and molecular levels have been insufficiently investigated in humans^6,54,56–58^. Yet, the available data suggest that unloading reduces the stiffness of the human Achilles and patellar tendons, mainly due to changes in material properties^54,56^. However, how patellar tendon stiffness is reduced without measurable change in tendon cross-sectional area after 14 days of unloading remains unclear^59,60^, but is likely due to a reduction in collagen cross-links^54,56,59^. While 90 days of immobilization did not induce MRI-based tendon atrophy^61^, spinal cord injury resulted in tendon degradation^62^, indicating the importance of duration of unloading on tendon atrophy. Given the above, and despite the limitations of analyzing tendons, ligaments, and connective tissue in vivo in humans, there is an urgent need to investigate how these tissues respond to prolonged periods of microgravity, particularly in terms of function and underlying mechanisms. An important general concept is that the research community has thus far treated the musculoskeletal system and even its forming tissues as individual components. However, the human body is a well-regulated system in which all organs and tissues are interdependent. Therefore, major efforts should be made to understand these interactions and to advance research that focuses especially on tissue interactions within the human body. Such investigations could be performed using a combination of ground-based analogs, animal and in vitro studies, and numerical models.

The ultimate goal for human spaceflight must be that no organ system fails, as this would likely trigger a chain reaction that would pose an increased health risk. Therefore, research analyzing specific crosstalk between the musculoskeletal system with immune, cardiovascular, neural, endocrine, and digestive systems should be performed. In addition, potential interactions between musculoskeletal and psychological health during spaceflight needs to be addressed.

#### Re-thinking countermeasures

Countermeasures to preserve musculoskeletal tissue mass and function comprise physical activity programs, nutrition, and drug interventions. While we focus here on countermeasures for the musculoskeletal system, a more comprehensive perspective on countermeasures is given in:

The ESA SciSpacEWhite Paper #15: Integrative and Countermeasures Approach (https://www.esa.int/Science_Exploration/Human_and_Robotic_Exploration/Research/The_SciSpacE_White_Papers).

Exercise countermeasures have been studied extensively, but the optimal program has yet to be found. While we (sort of) have a good idea of what is required for missions up to 6 months, longer missions may present an entirely new scenario, as, for example, the higher radiation exposure on exploration missions may exacerbate musculoskeletal degeneration. Future loads/protocols for countermeasures must take into account that all load-bearing tissues slowly deteriorate and thus lose the ability to withstand external loads. The difficulty is that these changes are not readily detectable with currently available in-flight instrumentation, particularly for tissues such as cartilage, bone, tendon, and ligaments, and are also unlikely to be noticed by crewmembers. Therefore, continued development of in-flight diagnostic tools and biomarkers and accelerated availability of novel in-flight diagnostic technologies seems essential. These alterations during exploratory missions also mean that countermeasure programs and loads that work at the beginning of a mission may not be as useful later or may even increase the risk of injury.

With respect to the musculoskeletal system, future countermeasure research should focus on preserving the mass and function of all musculoskeletal tissues, including tendons, ligaments, and connective tissue. It is also important to investigate the synergistic or additive effects of combinations of nutritional supplements, drugs, and exercise countermeasures. Additionally, given the potential benefits of hypergravity^63–65^, its efficacy should be further investigated^66–69^. Regarding increased radiation exposure, the effect of radiation protection agents needs to be investigated, and the interaction of radiation, microgravity, and countermeasures on the musculoskeletal system needs to be explored. The unique haracteristics of exploration missions will also require the development of treatment and support measures for musculoskeletal injuries, instrumentation that provides live feedback during countermeasures, and new countermeasure hardware complying with exploration mission requirements. Indeed, developing hardware that meets weight and volume requirements while providing high training loads to prevent muscle atrophy is a major challenge. In this context, it may be interesting to intensify research on the efficacy of low-load, high-volume resistance exercise programmes under spaceflight conditions, as these training regimes have been shown to promote as much muscle hypertrophy as high-load protocols on Earth^70,71^. While low-load, high-volume protocols require lighter hardware, further research is needed to better understand their effects on all tissues of the musculoskeletal system, as well as their influence on, for example, potential changes in energy expenditure that would alter nutritional aspects and thus food supply during exploration missions. Finally, future studies should consider psychological factors that may influence compliance with countermeasures.

### Post-flight – the forgotten period

There is a need to change the current approach, which focuses mainly on the early post-flight period, to investigate the recovery from spaceflight. In particular, after missions longer than two months, long-term follow-up of crewmembers is required to understand when full or partial recovery is achieved. These data can be used for model-based predictions of adaptation during longer space missions. One possible option for space agencies would be to consider calls for experiments that specifically target the post-flight period. Standardized research protocols during this phase could address points like tissue recovery with and without exercise, nutrition, or drug countermeasures. More specifically, it would be important to understand whether bone strength can recover, given the bone’s ability to adapt its morphology to loading conditions and achieve the necessary trabecular distribution to withstand the applied loads^72^, and whether anti-osteoporotic drugs in combination with exercise can aid in bone strength recovery^73^. Considering that some astronauts will fly more than once, it is important to understand potential differences in musculoskeletal tissue atrophy between single and multiple-flight crew and also whether the length of the recovery period on Earth between missions plays a role. For skeletal muscle, particularly muscle function, there are virtually no post-flight data beyond the first few months after landing. Here, the previously observed supercompensation of muscle mass after 90 days of bed rest^74^ should be investigated. As mentioned above for in-flight changes, the recovery of articular cartilage, tendons, ligaments, and connective tissue is unknown. Since it is important to maintain a good equilibrium in the recovery of the musculoskeletal system, specific periods of observation and testing should be standardized for the different tissues forming the musculoskeletal system.

### Other points of interest

There are other general points of interest that cut across all periods of an exploration mission.

In general, exploration missions will expose the crew to different types and doses of radiation (see white papers on Radiation). Studies in animal models show that the combination of unloading and radiation increases tissue degeneration^75–77^ and promotes unnoticed tissue pathologies (e.g., osteonecrosis). This should be verified in humans.

To better interpret the effects of long-term space missions, it may be useful to include standard control populations on Earth (e.g., astronaut back-ups and also general population) that are age- and sex-matched with the crew members. This would allow assessment of musculoskeletal adaptation in space relative to the naturally-occurring physiological changes on Earth. Furthermore, a core data set (e.g., similar to the ESA bed rest core data) to assess musculoskeletal adaptations during space flight could be an efficient tool to streamline research in the last decade of the ISS. In this context, the standardized collection of medical data would be of great benefit to the space life science community while saving resources. Another factor to consider is to increase efforts to facilitate multidisciplinary discussion among astronauts, physicians, crew surgeons, and technical personnel (increment science coordinators, payload specialists) along with space agencies and musculoskeletal researchers. Finally, space sciences need to open up to better statistics^78^, mathematical models, and digital twins to enable model-based prediction of musculoskeletal changes at the individual level.

### Priorities for human spaceflight

Preserving musculoskeletal function is essential for the success of exploration missions. We need to better understand what is required to keep the crew moving and what level of function is required to ensure mission success (e.g., landing and building infrastructure on Mars and the Moon, exploration, etc.). To accomplish this, it is very important to know the loads acting on the musculoskeletal system during a mission. Such information could be obtained in advance through modeling approaches and measured during the actual mission using specialized equipment. Another aspect crucial for monitoring musculoskeletal deconditioning in space is to find reliable biomarkers that are easy to measure and provide accurate information about all the tissues forming the musculoskeletal system. Such information should be made available in-flight in future exploratory-type missions.

### Benefits for Earth and industrial relevance

In general, space research offers a unique model to investigate the role of mechanical loading and acting gravitational forces on the musculoskeletal system. In this environment, we can accelerate and amplify the effects of immobilization and, consequently, the damage of a sedentary lifestyle to a biological system that has evolved to withstand mechanical loads during upright locomotion. Importantly, this research allows us to assess the isolated effects of immobilization without concomitant diseases, at least in younger-aged individuals typically tested in bed rest and other ground-based analog studies. The musculoskeletal changes observed in spaceflight research and the lessons learned about successful countermeasures can be applied to other tissue-deteriorating conditions in patients and be used to develop low-volume, low-weight devices and exercise protocols that may prove effective for home exercise training. Biological markers (molecular, functional, and imaging) that indicate critical musculoskeletal deconditioning before reaching a level of no return would also be relevant to clinical settings on Earth and could be useful in telemedicine situations. The fairly standardized setting of space research and the multidisciplinary approaches used allow for a detailed understanding of the interrelation of the musculoskeletal tissues, which is difficult to achieve in the clinical setting.

## Summary and conclusion

Exploratory missions are a big black box when it comes to the adaptation of the musculoskeletal system. It is unclear how well countermeasures can preserve tissue status on missions beyond 6 months and what a critical level of tissue mass and function would look like. To address these questions, it seems vital to use current missions to collect data from pre-, during-, and post-flight periods. This would provide important information to understand and ultimately offset musculoskeletal alterations during long-term spaceflight. In addition, understanding the kinetics of the different components of the musculoskeletal system (muscle, bone, tendon, ligament, connective tissue) in parallel with a detailed description of the molecular mechanisms driving these alterations appears to be the best approach to address potential musculoskeletal problems that future exploratory-mission crew will face. These research efforts should be accompanied by technical advances in molecular and phenotypic monitoring tools to provide real-time feedback for critical biomarkers of skeletal muscle deconditioning.

### Reporting summary

Further information on research design is available in the Nature Research Reporting Summary linked to this article.

## Supplementary information

Reporting Summary
